# The Mystery of Increased Hospitalizations of Elderly Patients

**DOI:** 10.3201/eid1405.080217

**Published:** 2008-05

**Authors:** Martin I. Meltzer

**Affiliations:** * Centers for Disease Control and Prevention, Atlanta, Georgia, USA

**Keywords:** Hospitalizations, elderly, pneumonia, community-acquired staphyloccal pneumonia, commentary

This issue of Emerging Infectious Diseases contains 2 articles that report increases in England in the incidence of hospitalizations for specific diseases, one on pneumonias (*1*) and the other on community-acquired staphylococcal diseases (*2*). Both articles report increases in disease among those >65 years of age.

Trotter et al. found a 20%–39% increase in pneumonia-related hospitalizations in persons >65 years from April 1997 through March 2005 (*1*). These researchers found that the older a person, the greater the risk for pneumonia-related hospitalization. Those older patients with coexisting conditions, as measured by a severity-of-illness scale (the Charlson Comorbidity Index), were also more likely to be hospitalized than those with no such recorded conditions. What is surprising, however, is that although the percentage of patients with moderate or severe coexisting conditions increased over time, the percentage who died within 30 days of admission decreased slightly. It is hard to think of an advance in medical science (e.g., a new “wonder drug”) within the period studied that would explain the stability of a 30-day mortality rate when hospitalizations among the frail elderly are increasing.

Hayward et al. (*2*) measured a 6-fold increase in hospitalizations for staphylococcal pneumonia and an ≈4-fold increase in hospitalizations for abscesses or cellulitis for patients >65 years of age from 1989–90 through 2003–04, (*2*). While these increases may be real and actual, they should be placed in some context by moving the focus from relative increases to actual numbers. As reported by Hayward et al., comparing 1989–90 to 2003–04, the age-adjusted rate of admission for abscesses, carbuncles, furuncles and cellulitis (all 4 combined) increased from 500 to 1,488 per million general population. For staphylococcal pneumonia, the rates increased from 2 to 12 per million (*2*). The rate for pneumonia (primary diagnosis) rose from 1,480 (1997–98) to 1,980 per million in 2004–05 (*1*).

These rates are not unexpected. In the United States in 2000, the rate of hospitalizations for staphylococcal diseases was ≈1,060 per million general population (*3**,**4*). Among those >65 years of age in 2002, the rate of hospitalizations for pneumonia (primary diagnosis) was equivalent to 2,100 per million all-ages general population (17,000/million >65 years) (*5*). (In the United States in 2005, there were 116,200 hospital discharges per million persons [*3*]. In 2000, a diagnosis of *S. aureus* infection was made in 9.13 of every 1,000 hospital discharges, or 0.913% [*4*].)

The rates and increases measured in England may seem large, but during the fiscal year 2004–05 the British National Health System recorded ≈12 million hospital “admission episodes,” including 6.8 million operations in England (*6*), a rate of ≈240,000 admissions per million population. Thus, while hospital staffs are very likely to see cases of staphylococcal disease and pneumonia, they will also see a much larger combined volume of patients with other diseases and conditions.

Being very busy, hospital staff may not actually notice the increases reported by Hayward et al. and Trotter et al. Hidden or not, the reported results are real, and the incidence of hospitalizations for certain diseases has clearly risen. However, as both sets of authors carefully point out, we do not know the cause of such increases. Both studies used the same dataset: the United Kingdom’s National Health Services hospital admissions database (Hospital Episodes Statistics). This is an administrative dataset, primarily designed to track data to allow administrators, politicians, and the public to gauge how well the healthcare system is responding to demand. For example, mean time waited until admitted to hospital is a key measure of the system’s ability to meet demand (in 2004–05, the mean wait time was 84 days [*7*]).

Such administrative databases do not, typically, contain the type of data that allow detailed analyses of epidemiologic risk factors. Both Hayward’s and Trotter’s articles contain extensive discussion of factors that could influence the measured increases in rates of hospitalizations.

Until we understand what caused these increases, designing interventions to target the root causes of such increases will be challenging. Although the increases should cause concern, and although the phrase “more research is needed” is often hackneyed and self-serving, in this situation it is warranted.
